# Prevalence of exclusive breastfeeding and associated factors among mothers in rural Bangladesh: a cross-sectional study

**DOI:** 10.1186/1746-4358-9-7

**Published:** 2014-05-29

**Authors:** Prakash Chandra Joshi, Mirak Raj Angdembe, Sumon Kumar Das, Shahnawaz Ahmed, Abu Syed Golam Faruque, Tahmeed Ahmed

**Affiliations:** 1 Department of Public Health, Central Institute of Science and Technology, Pokhara University, Kathmandu, Nepal; 2 James P. Grant School of Public Health, BRAC University, Dhaka, Bangladesh; 3 Centre for Nutrition and Food Security, International Centre for Diarrhoeal Disease Research, Bangladesh (icddr, b), Dhaka, Bangladesh

**Keywords:** Exclusive breastfeeding, Bangladesh, Prevalence, Factors, Predictors

## Abstract

**Background:**

Exclusive breastfeeding (EBF) means that the infant receives only breast milk for the first six months of life after birth. In Bangladesh, the prevalence of EBF remained largely unchanged for nearly two decades and was 43% in 2007. However, in 2011, a prevalence of 64% was reported, an increase by 21 percentage points. The reasons for this large change remain speculative at this point. Thus to investigate the issue further, this study was conducted. The objective was to assess the prevalence of EBF and associated factors among mothers having children aged 0–6 months in rural Bangladesh.

**Methods:**

A cross-sectional study was conducted in Mirzapur *Upazilla* (sub district) among 121 mothers of infants aged 0–6 months. Eligible mothers were identified and randomly selected using the demographic surveillance system’s computerized database that is updated weekly. A semi-structured questionnaire was used for interviews that inquired information on socio-demographic characteristics, obstetric, health service, breastfeeding related factors (initiation of breastfeeding, prelacteal feeding and colostrum feeding) and economic factors. EBF prevalence was calculated using 24 hour recall method. In multivariate analysis, a logistic regression model was developed using stepwise modeling to analyze the factors associated with EBF.

**Results:**

The prevalence of EBF in the last 24 hours preceding the survey was 36%. Bivariate and multivariate analysis revealed no significant association between EBF and its possible predictors at 0.05 level of alpha. However, there was some evidence of an association between EBF and having a caesarean delivery (OR = 0.47, 95% CI: 0.21, 1.06). In multivariate analysis, type of delivery: caesarean (AOR = 0.45, 95% CI: 0.19, 1.03) and wealth quintile: richer (AOR = 2.40, 95% CI: 0.94, 6.16) also showed some evidence of an association with EBF.

**Conclusion:**

The prevalence of EBF in Mirzapur (36%) is lower than the national figure (64%). Prelacteal feeding was not uncommon. These findings suggest that there is a need for breastfeeding support provided by health services. Hence, promotion of EBF during the first six months of life needs to be addressed and future breastfeeding promotion programmes should give special attention to those women who are not practicing EBF.

## Background

Breastfeeding is a well established and recommended intervention for the improvement of child nutrition. Studies have demonstrated that it reduces deaths in infants and young children [1-3]. It is one of the most important factors for growth and development of infants and is globally endorsed as being the best for any neonate [4]. The World Health Organization (WHO) recommends the practice of exclusive breastfeeding (EBF) of infants for the first six months of life after birth. EBF means that the infant receives only breast milk. No other liquids or solids are given, not even water, with the exception of oral rehydration salt solution, or drops/syrups of vitamins, minerals or medicines [5].

Throughout the world, in developed and developing countries alike, inappropriate feeding of infants leading to their poor nutrition is a significant problem affecting socio-economic progress in general [6]. Suboptimum breastfeeding was responsible for 11.6% of all child deaths in 2011 [7]. Universal (90%) coverage of breastfeeding is estimated to prevent around 13% of all deaths among children under five years of age in low and middle income countries [3]. However, globally only half of infants under 1 month of age and 30% of infants aged 1–5 months are exclusively breastfed [7].

Previous studies have reported several predictors of EBF. Differences are evident not just between countries but also within the same country. Several factors have been shown to be associated with EBF: variations between urban and rural areas, infant’s age, mothers’ employment status and education level, knowledge about good breastfeeding practices, occupation, monthly household income, mothers’ smoking status, socio-economic position, prelacteal feeding, parity, positive attitudes towards EBF, intent to exclusively breastfeed before delivery, timely initiation of breastfeeding, mode of delivery, infant’s birth weight, health system practices, discarding colostrum and community beliefs [8-14].

In Bangladesh, the prevalence of EBF has remained largely unchanged for nearly two decades. It was around 45% in 1993–94 and 1999–2000 [15,16], then declined to 42% in 2004 [17] and was 43% in 2007 [18]. However, in 2011, a prevalence of 64% was reported, an increase of 21 percentage points [19]. The reasons for this large change remain speculative at this point. Thus to investigate the issue further, this study was conducted in a rural sub district where EBF prevalence had not been investigated by any prior studies. The objective was to assess the prevalence of EBF and associated factors among mothers having infants aged 0–6 months in rural Bangladesh.

## Methods

A cross-sectional study was carried out in Mirzapur *Upazilla* (Sub district) of Tangail district in rural Bangladesh among mothers having infants aged 0–6 months. The rationale for selecting the site was: Tangail is ranked 37^th^ for the timely initiation of breastfeeding out of 64 districts, and Mirzapur sub-district under Tangail district is ranked 79^th^ for timely initiation of breastfeeding out of 483 sub-districts of Bangladesh [20]. Thus, Mirzapur is a typical sub-district of Bangladesh. It is neither a ‘high performing sub-district’ nor a ‘low performing sub-district’. In addition, there were no focused nutrition interventions including promotion of breastfeeding programmes going on in that area. A baseline prevalence of 43% [18], margin of error 10%, with 95% CI was used to calculate the sample size. Further a design of effect 1.2 was added [21] which resulted in a sample size of 121. Taking into account a non-response of 25% the final sample size was calculated to be 151.

**Figure 1:**
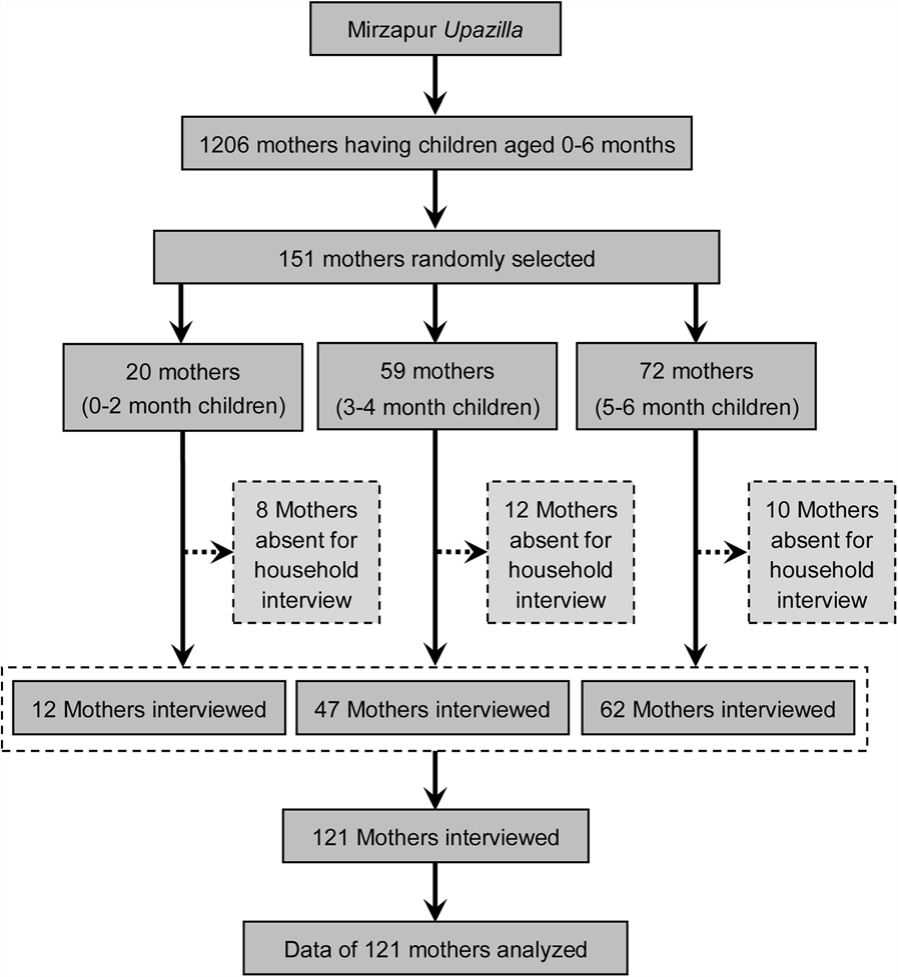
Sampling profile.

The list of mothers having infants aged 0–6 months was obtained from the demographic surveillance systems (DSS) computerized database that is updated on a weekly basis by field staffs of International Centre for Diarrhoeal Disease Research, Bangladesh (icddr, b). The list comprised of 1206 mothers having infants aged 0–6 months. From that list, required number of mothers in each stratum (0–2 months, 3–4 months and 5–6 months) was proportionately calculated and randomly selected using SPSS (version 20) software (Figure 1). A total of 151 mothers were randomly selected. The households were then identified and mapped for ease of data collection. Out of 151 mothers, 30 were absent during interview and were not followed up thereafter. In the end, we interviewed and analyzed data from 121 mothers: 12 mothers of 0–2 month old children, 47 mothers of 3–4 month old children and 62 mothers of 5–6 month old children. Data were collected in November 2012 by using pre-tested semi-structured questionnaire that inquired information on socio-demographic characteristics, obstetric, health service and breastfeeding related factors (initiation of breastfeeding, prelacteal feeding and colostrum feeding) and economic factors. Questions related to EBF were asked using 24 hour recall method. Survey questionnaires were administered in native language (*Bangla*) of the respondents.

Data were entered in SPSS (version 20) and analyzed using SPSS (version 20) and Stata (version 12). The outcome variable was EBF as per WHO definition [5]. Wealth index was constructed using household asset data [18]. It comprised of ownership of several consumer items. Principal component analysis was used to generate and assign weight (factor score) to each asset. The asset scores for individual household were then computed and divided into quintiles from one (poorest) to five (richest). Univariate analysis was carried out for all variables to describe their characteristics. In bivariate analysis, crude odds ratio (OR) with 95% CI and Chi-square test or Fisher’s exact test (as appropriate) were calculated to identify associations between categorical variables. In multivariate analysis, a logistic regression model was developed to identify the associations between the dichotomous outcome variable: EBF (yes/no) and potential predictors. The final model was generated by stepwise backward elimination procedure and adjusted odds ratios (AOR) were calculated with 95% CI. Thus, the final model had only those predictors that showed some evidence of association with the outcome variable.

Ethical approval was obtained from the ethical review committee at James P. Grant School of Public Health, BRAC University, Dhaka. Prior to interview, written informed consent was obtained from the mothers. In cases where the mother was illiterate, verbal consent was taken from the mother following a detailed description of the study protocol. The interviewer also signed a statement for verifying that he had provided the necessary information written in the informed consent form and that each respondent had consented to participate in the study. All data were coded to remove identifying information and secure confidentiality. Respondents were not compensated for participation in the study.

## Results

A total sample of 121 mothers having infants aged 0–2, 3–4 and 5–6 months were interviewed. The background characteristics of the respondents are presented in Table 1. There were more male (51%) than female (49%) children. Among 121 children, 36% were first born. Mothers’ age ranged from 16 to 40 years with a mean of 25 ± 5.2 years (information not included in Table 1). Majority of the mothers lived in a joint family (56%) and had primary education (78%). Only 6% of the mothers were employed. Sixty four percent of fathers were educated up to primary level. One fifth of the families belonged to the poorest wealth quintile and another one fifth belonged to poorer wealth quintile.

**Table 1:** Characteristics of mothers, children and breastfeeding factors

**Characteristics (N = 121)**	**n**	**%**	**95% CI**
Sex of the child			
Male	69	51.2	42.2, 60.3
Female	59	48.8	39.7, 57.8
Birth order of the child			
First born	44	36.4	27.7, 45.1
Second and above	77	63.6	54.9, 72.3
Mothers’ age^#^ (years)			
≤18	12	9.9	4.5, 15.3
>18	109	90.1	84.7, 95.5
Mothers’ education			
Illiterate (no education)	13	10.7	5.1, 16.3
Primary education (completed grade 5)	94	77.7	70.2, 85.2
Secondary or higher education (completed grade 10 and higher)	14	11.6	5.8, 17.4
Mothers’ employment status			
Housewife	114	94.2	9.0, 9.8
Working outside home	7	5.8	1.6, 10.0
Fathers’ education			
Illiterate	17	14.0	7.8, 20.3
Primary education	78	64.5	55.8, 73.1
Secondary education	26	21.5	14.1, 28.9
Type of family			
Nuclear	53	43.8	34.8, 52.8
Joint and others	68	56.2	47.2, 65.2
Wealth quintile			
Poorest	25	20.7	13.3, 28.0
Poorer	26	21.5	14.1, 28.9
Middle	23	19.0	11.9, 26.1
Richer	23	19.0	11.9, 26.1
Richest	24	19.8	12.6, 27.0
Breastfeeding education received			
Yes	63	52.1	43.0, 61.1
No	58	47.9	38.9, 57.0
No. ANC visits			
No visits	17	14.0	7.8, 20.3
1-3	82	67.8	59.3, 76.2
≥4	22	18.2	11.2, 25.2
Place of delivery			
Institutional	69	57.0	48.1, 66.0
Non-institutional	52	43.0	34.0, 5.9
Type of delivery			
Normal	78	64.5	55.8, 73.1
Caesarean	43	35.5	26.9, 44.2
Assistance during delivery			
Doctor	54	44.6	35.6, 53.6
Nurse/sisters and others	15	12.4	6.4, 18.4
Traditional birth attendants	52	43.0	34.0, 51.9
Initiation of breastfeeding			
≤1 hour	56	46.3	37.3, 55.3
>1 hour	65	53.7	44.7, 62.7
Received colostrum			
Yes	116	95.9	92.3, 99.5
No	5	4.1	0.5, 7.7
Prelacteal feeding			
Yes	23	19.0	11.9, 26.1
No	98	81.0	73.9, 88.1
Prelacteal feeds*			
Other milk than breast milk	2	8.7	NA
Sugar/glucose water	9	39.1	NA
Infant formula	5	21.7	NA
Honey	3	13.0	NA
Drop	7	30.4	NA

Note: Child refers to the reference child aged 0–6 months.

^#^Dichotomization based on legal age of marriage in Bangladesh.

*Multiple responses.

NA: Not Applicable.

**Figure 2:**
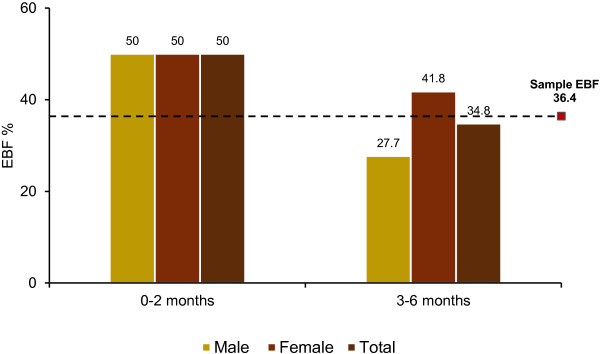
Prevalence of exclusive breastfeeding by age and sex.

Majority (52%) of the mothers reported that they had received breastfeeding education during their last pregnancy and 18% percent of mothers had sufficient (≥4) antenatal care visits. Majority (57%) of the mothers had delivered in a health institution during their last pregnancy. Most of these were normal deliveries (65%) assisted by doctors (45%). More than half (54%) of the children were initiated breastfeeding after one hour, and a large percentage (96%) received colostrum. Nineteen percent of the children had received prelacteal feeds out of which most of the children (40%) were given sugar/glucose water.The prevalence of EBF in the last 24 hours preceding the survey was 36%. Among older infants (3–6 months) EBF prevalence was 35% and among younger infants (0–2 months) it was 50% (Figure 2). Among older infants the prevalence was higher among females (42%) than males (28%) but the difference was not statistically significant. Most of the mothers reported that the main reason for not practicing EBF was inadequate secretion of breast milk (64%). Other reasons were: care taker fed the child with food other than breast milk unknowingly (7%), children were unable to suck the breast (7%), and remaining 23% of the mothers mentioned several other reasons which included: being influenced by a neighbour who does not exclusively breastfeed her child, lack of nutritious food to the mother causing inadequate secretion of breast milk and illness of the mother.

In bivariate analysis, none of the factors were associated with EBF at 0.05 level of alpha (Table 2). However some evidence of an association was observed with type of delivery (OR = 0.47, 95% CI = 0.21, 1.06). Mothers who delivered by caesarean section had lower odds of EBF than those who delivered normally. Likewise in multivariate analysis no any factors showed significant association at 0.05 level of alpha, but again there was some evidence of association with type of delivery: caesarean (*p* = 0.067)and wealth quintile: richer (*p =* 0.512) (Table 3). Keeping all factors constant mothers who delivered by caesarean section had lower odds of EBF than those who delivered normally (AOR = 0.45, 95% CI = 0.19, 1.03). The mothers from households belonging to richer wealth quintile had 2.4 times higher odds of EBF compared to those belonging to poorest wealth quintile keeping all factors constant (AOR = 2.40, 95% CI = 0.94, 6.16).

**Table 2:** Bivariate analysis of factors associated with exclusive breastfeeding

**Variables**	**Exclusive breastfeeding**				**OR (95% CI)**	***p*-value**
	**Yes (n = 44)**		**No (n = 77)**			
	**n**	**%**	**n**	**%**		
Sex of the child						
Male	19	30.6	43	69.4	1.00	0.180^a^
Female	25	42.4	34	57.6	1.66 (0.79, 3.51)	
Birth order of the child						
First born	15	34.1	29	65.9	1.00	0.694^a^
Second and above	29	37.7	48	62.3	1.17 (0.54, 2.54)	
Mothers’ age^#^ (years)						
≤18	4	33.3	8	66.7	1.00	1.000^b^
>18	40	36.7	69	63.3	1.16 (0.33, 4.09)	
Mothers’ education						
Illiterate	6	46.2	7	53.8	1.00	0.437^a^
Literate	38	35.2	70	64.8	0.63 (0.20, 2.02)	
Mothers’ employment status						
Housewife	40	35.1	74	64.9	1.00	0.255^b^
Working outside home	4	57.1	3	42.9	2.47 (0.52, 11.57)	
Fathers’ education						
Illiterate	8	47.1	9	52.9	1.00	0.323^a^
Literate	36	34.6	68	65.4	0.59 (0.21, 1.67)	
Type of family						
Nuclear	22	41.5	31	58.5	1.00	0.299^a^
Joint and others	22	32.3	46	67.7	0.67 (0.32, 1.42)	
Wealth quintile						
Poorest	9	36.0	16	64.0	1.00	0.512^a^
Poorer	8	30.8	18	69.2	1.27 (0.39, 4.06)	
Middle	7	30.4	16	69.6	1.29 (0.38, 4.30)	
Richer	12	52.2	11	47.8	1.52 (0.16, 1.64)	
Richest	8	33.3	16	66.7	1.13 (0.35, 3.65)	
Breastfeeding education received						
Yes	24	38.1	39	61.9	1.00	0.680^a^
No	20	34.5	38	65.5	1.17 (0.56, 2.46)	
No. ANC visits						
No visits	6	35.3	11	64.7	1.00	0.870^a^
1-3	31	37.8	51	62.2	0.90 (0.30, 2.67)	
≥4	7	31.8	15	68.2	0.63 (0.16, 2.40)	
Place of delivery						
Non-institutional	22	42.3	30	57.7	1.00	0.238^a^
Institutional	22	31.9	47	68.1	0.64 (0.30, 1.35)	
Type of delivery						
Normal	33	42.3	45	57.7	1.00	0.067^a^
Caesarean	11	25.6	32	74.4	0.47 (0.21, 1.06)	
Initiation of breastfeeding						
≤1 hour	24	42.9	32	57.1	1.00	0.168^a^
>1 hour	20	30.8	45	69.2	0.59 (0.28, 1.25)	
Received colostrum						
Yes	43	37.1	73	62.9	1.00	0.652^b^
No	1	20.0	4	80.0	2.36 (0.25, 21.77)	
Prelacteal feeding						
Yes	9	39.1	14	60.9	1.00	0.759^a^
No	35	35.7	63	64.3	1.16 (0.45, 2.94)	

^a^Chi-square test (2-tailed).

^b^Fisher’s exact test.

^#^Dichotomization based on legal age of marriage in Bangladesh.

**Table 3:** Logistic regression model for predictors of exclusive breastfeeding

**Variables**	**AOR**^**#**^	**95% CI**	**p-value**
Type of delivery			
Normal	1.00		
Caesarean	0.45	0.19, 1.03	0.058
Wealth quintile			
Poorest	1.00		
Richer	2.40	0.94, 6.16	0.069

Covariates adjusted were type of family, mother’s age, mother’s education, mother’s employment status, father’s education, sex of the child, birth order of the child, age of the child, number of ANC visits, place of delivery, type of delivery, breastfeeding education, initiation of breastfeeding, colostrum feeding, prelacteal feeding and wealth.

^**#**^Adjusted Odds Ratio.

## Discussion

EBF is the best recommended infant feeding method for the first six months of life and has a protective effect against child morbidity and mortality [22]. But it has not yet been universally practiced and the reduction in the rate of EBF is taken as a serious problem, especially in developing countries [22]. In the present study, prevalence of EBF was lower (36%), compared to national level (64%) [19]. The Bangladesh Demographic and Health Survey (BDHS) results were from the whole country which includes nutrition intervention and non-intervention districts. In comparison, our study was conducted in a non-intervention district which did not have any nutrition and breastfeeding promotion programmes. This might be one of the reasons why prevalence of EBF was lower in our study. The BDHS has attributed the higher prevalence of 64% to the inclusion of greater number of young infants in their sample [19]. Mothers are more inclined to exclusively breastfeed their children when they are young and this could result in over estimation of EBF prevalence. In contrast, the number of young infants (0–2 months) in our study was too low. As a result we could not make direct comparisons with the BDHS result. Thus we have disaggregated the prevalence by age and reported for older age groups as well. Additionally, other reasons for low prevalence in our study may include lack of support from family members, lack of advice from health staffs during ANC visits and deliveries in non baby friendly institutions. The baby friendly hospital initiative was established by WHO and UNICEF in 1991 as a hospital-based intervention to increase breastfeeding rates. In baby friendly hospitals breastfeeding is supported, practiced, protected and promoted [23].

Chudasama et al. reported prevalence of EBF to be 37% in Gujrat, India [24]. Another study from India among rural women in Tamil Nadu showed the prevalence to be 34% [25] which is similar to our study. Results may be similar because both the study sites were rural and both countries have similar type of cultural practices. Statistically significant differences were not observed between those who exclusively breastfed and those who do not with respect to variables that are considered to be supportive for breastfeeding such as maternal education, number of ANC visits, breastfeeding support and education, and place of delivery which was similar to a study conducted in India [26].

Children are more likely to receive prelacteal feeds when they are born at home and when the birth is not assisted by a health professional [19]. Over the years there has been a decline in the percentage of prelacteal feed at the national level - 62% in 2007 [18] and 39% in 2011 [19]. This could be explained by the decrease in the percentage of home deliveries - 85% in 2007 [18] and 71% in 2011 [19]. This might as well apply to our study where prelacteal feeding and home deliveries both are comparatively low.

Even though the study site was a fairly typical low-income upazilla, the presence of a large charity hospital-"Kumudini Hospital" meant that services were provided free of cost to all those who were unable to afford medical care; as a result of which a large number of residents sought health care services from the hospital. This might be the reason why many deliveries were institutional and assisted by doctors or nurses. On the contrary, at the national level 71% of the births were delivered at home [19].

Type of delivery was associated with EBF status. The mothers who delivered by caesarean section had lower odds of EBF than those who delivered normally. Ideally, all hospitals are required to practice ten steps to successful breastfeeding recommended by WHO [27] but it is seldom practiced. This could be one of the reasons why mothers who delivered by caesarean section did not practice EBF. As a result, the baby may be fed prelacteals even before breastfeeding is initiated for the first time. Furthermore, the mother and her family often think that breast milk itself is not sufficient and infant formula is necessary which is easily available around the hospital premises. A study from China [28] has reported that after surgery (c-section) mothers feel pain around the incision area and there is difficulty in movement because of catheterization and intravenous lines. The authors of this study argued that this could be a reason for non EBF. Similar explanation could also be valid for our study. Researchers from Nepal have also reported low EBF rates among mothers who delivered by caesarean section [29]. Further studies are needed to better understand the influence of caesarean section on EBF.

We found that the mothers from households belonging to richer wealth quintile had higher odds of EBF than those belonging to poorest wealth quintile. In contrast, other studies [17,30] have reported prevalence of EBF to be higher among children belonging to poorest wealth quintile. Mothers belonging to richer wealth quintile may have better education level, easier access to media and health services which may have increased their awareness and made them relatively more conscious about EBF.

There may be an intrinsic relation between EBF and its determinants but the present study did not aim to discuss those in detail. The present study alerts us that even though there is no relation with its determinants, the prevalence of EBF is very low which needs to be addressed. Further studies are necessary to investigate several other factors which are known to have relation with EBF. In addition, other psychological factors which include emotional symptoms, peer problems, or social behavior, parent's marriage or the mother's satisfaction with her relationship with her partner or child may be explored. Anthropological questions including the influence of conflict, work place/condition, culture, tradition, and environment need to be answered.

In our study, there was a non-response of 25%. Several factors could be held accountable for it. First, the respondents who were not available had recently migrated from the study site. Second, there was a culture of visiting maternal home (respondent's parents’ home) soon after the birth of the child. Third, either the respondent or her child was hospitalized due to some illness. Further limitations of this study include the use of 24-hour recall data for the calculation of EBF which may be inadequate. Despite the limitation, the strength of the study was the use of unbiased sample size with stratification, appropriate sampling design and analysis plan and selection of a study area where there was no nutrition intervention which resulted in unbiased estimates. If the study would have been conducted in a nutrition intervention area then the information collected would have been greatly influenced by the programme activities. An additional strength of our study is that it was conducted in a DSS site of icddr,b where the use of a computerized database of households with eligible respondents for sampling helped to increase the internal validity. The database was well maintained and updated weekly.

## Conclusion

The prevalence of EBF (36%) in Mirzapur is lower than the national figure (64%). Prelacteal feeding was not uncommon. These findings indicate that the breastfeeding support provided by health services is weak. Further, there were no nutrition interventions and breastfeeding promotional activities going on in that area. Hence, promotion of EBF during the first six months of life and continuation of breastfeeding after six months along with appropriate complementary feeding, and focus on the factors associated with EBF need to be addressed. Future breastfeeding promotion programmes by the government and partners should give special attention to those women who are not practicing EBF.

## Competing interests

The authors declare that they have no competing interests.

## Authors’ contributions

PCJ participated in the conceptualization and design of the study, performed data collection and participated in analysis and preparation of the manuscript. MRA participated in the design and data collection of the study, performed statistical analysis and drafted the manuscript. SKD and SA participated in the design of the study, helped collecting the data and preparation of the manuscript. ASGF participated in the design of the study, helped collecting and analyzing the data and draft the manuscript. TA conceived and supervised the study, helped interpret the results and made critical revisions to the paper. All authors read and approved the final manuscript.
